# How Some Girls Live

**Published:** 1888-10

**Authors:** 


### HOW SOME GIRLS LIVE.

They go to bed at night and fall into a sort of stupor; why not? Is there one breath of fresh air in their sleeping box? Do they ever, except in the heat of summer, have so much as a crack of the window open! If there is a fireplace in their room or a stovepipe hole, don't they close it up as tightly as they can? No wonder it is so hard to wake up in the morning. I can hear them groan, and moan, and yawn, and scold now, at the imperative summons to get up. And what do they find on their breakfast table? Sweet fried cakes, something in the shape of meat, generally fried, potatoes either fried or stewed, hot coffee and probably "griddle-cakes," fried of course. Now, I am not going on a crusade against the frying-pan, for it has its uses, but when I see a girl sit down at the breakfast table with dull eyes, a sallow face, a listless manner, and proceed to make that early meal of strong coffee, sweetened cakes, fried pork, and potatoes, with a sequence of griddle cakes liberally buttered and drowned in molasses, I feel like shutting her up for a week's starvation on bread and water.

Then there is dinner ; tough meat, baked vegetables, pie, any kind of a pie with a crust either tough or sandy, tasting strongly of lard and filled with things most convenient. A favorite pie in our country homes is constructed of sliced lemon, flour, and molasses, baked in a mass as unfit for the human stomach as a stewed rubber overshoe.

Tea-time brings cakes of various sorts, probably more pie, cheese, fruit preserved, and so ill done it is fermented, or canned fruit which is comparatively harmless, strong tea and hot biscuit. Repasts fit for

"A Cassowary,
On the plains of Timbuctoo."

Then to begin the day again. After breakfast they run up stairs and spread up their bed with all the exhalations of their bodies during the night still imprisoned in it. At bed-time they slip into their unaired beds after hanging the dresses they have worn eight or ten hours in that tight-shut closet, and repeat the experience of the night before.

"Now they have sown the seed,
What will the harvest be ?"

If it is winter, a heavy cold ; the misused lungs forced to breathe over and over, the air has no vitality in it, air that is absolutely noxious, become congested more or less, and they begin to cough and sneeze. If they have scrofula hidden in their constitutions, and how few people have not, the harvest of this planting will be bronchitis or consumption.

The next crop is dyspepsia ; they put into that delicate organ, the human stomach, already ingested by the hard labor of its next neighbor, the lungs, and weakened by the slow circulation of vitiated blood, vitiated by the bad air, a mass of indigestible stuff that they call food ; at first they do not notice any special effect ; they are young and strong and can bear a good deal of physical misfortune without much trouble, but after a time food begins to distress them, life gets very tiresome, they have acid tastes in their mouths, heart-burn, flatulence. Yes, I know these are unpleasant things to talk of, but they are a great more unpleasant to have.

This is a bad crop ; it realizes the primeval curse. "Thorns and briers shall it bring forth unto thee."

So they begin to "diet ;" but there is a harvest from that corset that abrogates the good of "diet" !

"Oh!" say a host of voices, one after another, "I can't live without my corset! the minute I leave it off I am as weak as a kitten; all gone I can hardly sit up."

They have told the story now! They have disabled themselves; they have ruined the wonderful work of God in their bodies, and the result, the harvest is fearful; their interior organs are all forced out of place, crowded, weakened, congested. Then to all this they add high-healed shoes. Possibly they do not know that the most delicate organs of their frames are only kept in place by muscular attachments, hung, as it were, on the edge of those wonderful muscles that do the work of life; when they wear high heels they throw these organs forward where they do not belong; they produce displacement. Do you know that means one kind of torture that grows into ulceration; another out of anguish; and their lives, their usefulness, their comforts are ruined.

Now, when these are gone, what can money do for them? What help is marriage? A sickly wife, a helpless mother! Will clothes, however gorgeous, alleviate a backache? or assuage dyspepsia? Will education do their ailments one particle of good?—*Phrenological Journal.*

